# 324. Detection of Antibiotic Resistance Genes and Associated Genera Direct from Blood Samples by Multiplex PCR and Next Generation Sequencing

**DOI:** 10.1093/ofid/ofac492.402

**Published:** 2022-12-15

**Authors:** Daniel Gamero, Kelly Dabrowski, Analisa DeVito, Roger Smith, Jessica L Snyder

**Affiliations:** T2 Biosystems, Inc, Lexington, Massachusetts; T2 Biosystems, Inc, Lexington, Massachusetts; T2 Biosystems, Inc, Lexington, Massachusetts; T2 Biosystems, Inc, Lexington, Massachusetts; T2 Biosystems, Inc, Lexington, Massachusetts

## Abstract

**Background:**

While blood culture and genotypic or phenotypic testing is standard procedure for identification of sepsis pathogens and resistance, blood culture negative sepsis occurs in 40-60% of patients. A culture independent diagnostic can identify pathogens that do not grow in culture and may provide a more sensitive indicator to blood stream infections. Here we discuss an assay that combines multiplex PCR with next generation sequencing (NGS) to detect resistance genes and pathogenic genera directly from whole blood. These genes include *mecA*, *mecC*, *vanA, vanB*, *bla*_KPC_, *bla*_OXA-48_ family, *bla*_NDM_, *bla*_VIM_, *bla*_IMP_, *bla*_CMY-2_, *bla*_DHA_, and *bla*_CTX-M-14_ and *bla*_CTX-M-15_ groups.

**Methods:**

K_2_EDTA whole blood samples were spiked with low titers of antimicrobial resistant bacterial isolates. Sample processing involved chemical lysis of red blood cells, concentration and mechanical lysis of pathogen cells, and multiplex amplification of resistance genes and 16S rRNA gene. A synthetic DNA oligo was added at a fixed concentration as a read control. Samples were sequenced on Illumina platforms. NGS data was analyzed using a bioinformatics pipeline to process and match sequences to curated databases with BLAST. The number of reads per sequence was normalized by the number of reads for the read control, resulting in a read-control-normalized (RCN) value that could be used to establish positivity cutoffs.

**Results:**

Resistance genes in bacterial pathogen spiked samples were identified at titers ≤ 10 CFU/mL. Owing to a unique dual indexing strategy, cross-reactivity was absent or low enough that cutoffs could be set using RCN values without reducing sensitivity. The expected genus was also detected at ≤ 10 CFU/mL for all samples; however, genera that may be representative of reagent or environmental contamination were observed at levels higher than a universal RCN cutoff in some samples.

**Conclusion:**

We have shown that this NGS assay can detect 13 antimicrobial resistance genes with high sensitivity and specificity direct from blood. Bacterial genera associated with these genes were detected by this assay. As there is a risk of detection of contaminating species with a 16S target, further research into low DNA reagents or environmental controls may be required.

**Disclosures:**

**Daniel Gamero, BS**, T2 Biosystems, inc: WO2018213641A8, EP3861013A1, WO2020055887A1, WO2020252084A1, WO2020257691A1|T2 Biosystems, inc: Stocks/Bonds **Kelly Dabrowski, M.S.**, T2 Biosystems, Inc: Stocks/Bonds **Analisa DeVito, B.S.**, T2 Biosystems, Inc: Stocks/Bonds **Roger Smith, Ph.D.**, T2 Biosystems, Inc: US20180188195A1, EP3849938A1,WO2020257691A1, WO2020252084A1|T2 Biosystems, Inc: Stocks/Bonds **Jessica L. Snyder, Ph.D.**, T2 Biosystems, Inc: CA3062882A1,US20180171388A1,WO2017180745A1 , EP3861013A1 , AU2020296183A1, WO2020055887A1, WO2020252084A1|T2 Biosystems, Inc: Stocks/Bonds.

